# Oral neuromuscular training in patients with dysphagia after stroke: a prospective, randomized, open-label study with blinded evaluators

**DOI:** 10.1186/s12883-020-01980-1

**Published:** 2020-11-07

**Authors:** Patricia Hägglund, Mary Hägg, Eva Levring Jäghagen, Bengt Larsson, Per Wester

**Affiliations:** 1 Department of Odontology, Oral and Maxillofacial Radiology, Umeå University, SE-90187 Umeå, Sweden; 2 Umeå Stroke Center, Department of Public Health and Clinical Medicine, Umeå University, Umeå, Sweden; 3 Speech & Swallowing Centre, Department of Otorhinolaryngology, Hudiksvall Hospital, Hudiksvall, Sweden; 4 Centre for Research & Development, Uppsala University/County Council of Gävleborg, Gävle, Sweden; 5 Department of Radiology, Hudiksvall Hospital, Hudiksvall, Sweden; 6 Department of Clinical Sciences, Karolinska Institutet, Danderyd University Hospital, Stockholm, Sweden

**Keywords:** Swallowing disorder, Oral screen, Rehabilitation, Swallowing capacity, Videofluoroscopy, Radiology

## Abstract

**Background:**

Oral and pharyngeal swallowing dysfunction are common complications in acute stroke patients. This primary aim of this study was to determine whether oral neuromuscular training improves swallowing function in participants with swallowing dysfunction after stroke. A secondary aim was to assess how well results of the timed water-swallow test (TWST) correspond with swallowing dysfunction diagnosed by videofluoroscopy (VFS).

**Methods:**

This was an intention-to-treat two-centre prospective randomized open-label study with blinded-evaluators (PROBE) design. At 4 weeks after stroke onset, participants with swallowing dysfunction were randomized to 5 weeks of continued orofacial sensory-vibration stimulation with an electric toothbrush or additional oral neuromuscular training with an oral device (Muppy®). Participants were examined with TWST, a lip-force test, and VFS before (baseline), after 5 weeks’ treatment (the end-of-treatment), and 12 months after treatment (follow-up). The baseline VFS results were compared with the TWST results. The primary endpoint was changes in swallowing rate assessed using TWST, from baseline to the end of training and from baseline to follow-up based on intention-to-treat analyses. The secondary endpoint was the corresponding changes in lip-force between baseline, the end of treatment, and follow-up.

**Results:**

The participants were randomly assigned as controls (*n* = 20) or for intervention with oral neuromuscular training (*n* = 20). After treatment, both groups had improved significantly (intervention, *P* < 0.001; controls, *P* = 0.001) in TWST but there was no significant between-group difference in swallowing rate. At the 12-month follow-up, the intervention group had improved further whereas the controls had deteriorated, and there were significant between-group differences in swallowing rate (*P* = 0.032) and lip force (*P* = 0.001). A TWST < 10 mL/sec at baseline corresponded to VFS-verified swallowing dysfunction in all assessed participants.

**Conclusion:**

The 5-week oral neuromuscular training improved swallowing function in participants with post-stroke dysphagia compared with the controls 12 months after intervention, but there was no between-group difference in improvement immediately after treatment. TWST results corresponded with VFS results, making TWST a feasible method for identifying persons with swallowing dysfunction after stroke. Larger randomized controlled trials are required to confirm our preliminary positive long-term results.

**Trial registration:**

Retrospectively registered at ClinicalTrials.gov: NCT04164420. Registered on 15 November 2019.

**Supplementary Information:**

The online version contains supplementary material available at 10.1186/s12883-020-01980-1.

## Background

**Fig. 1:**
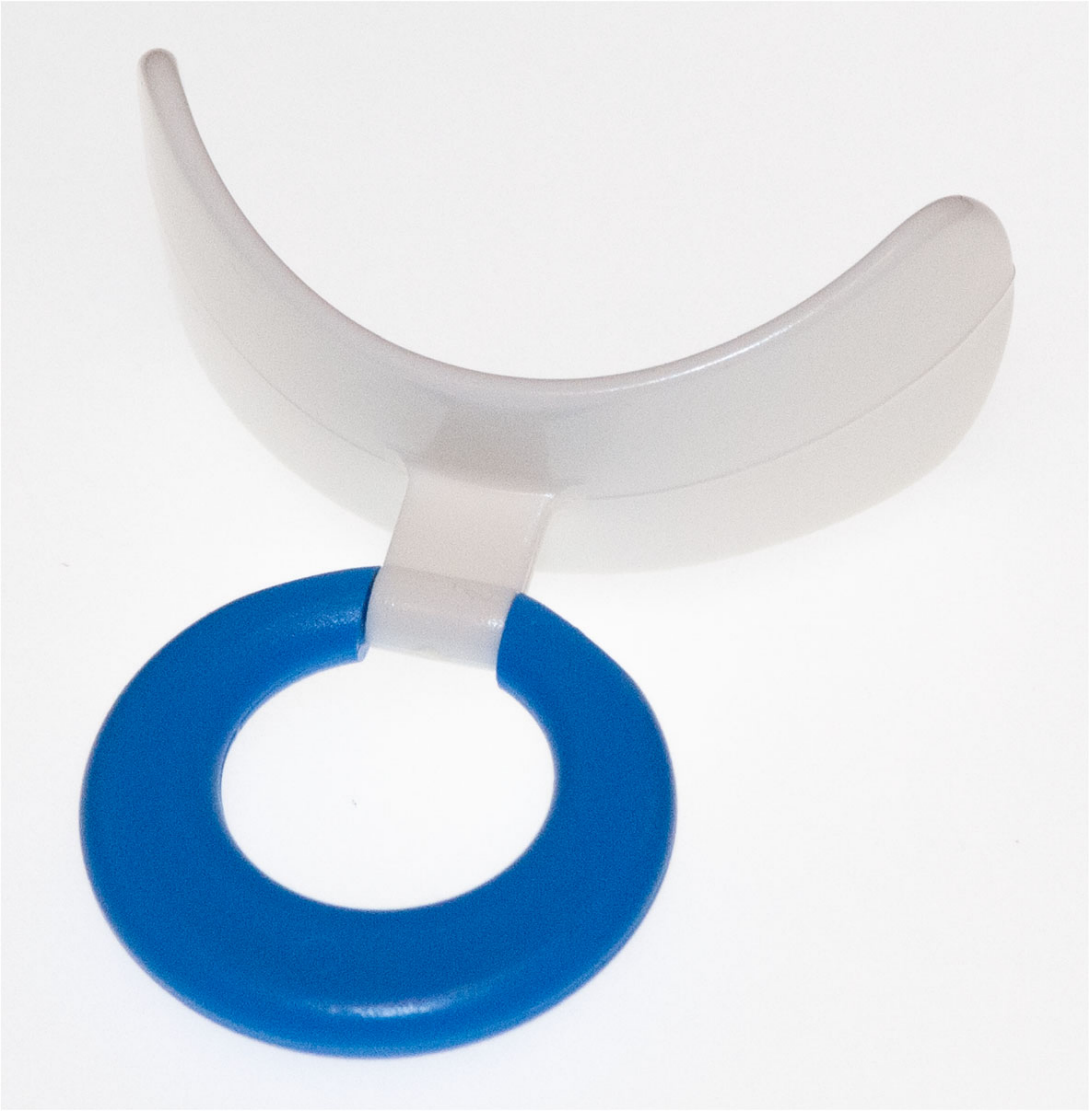
The oral device (Muppy®) (own image)

**Fig. 2:**
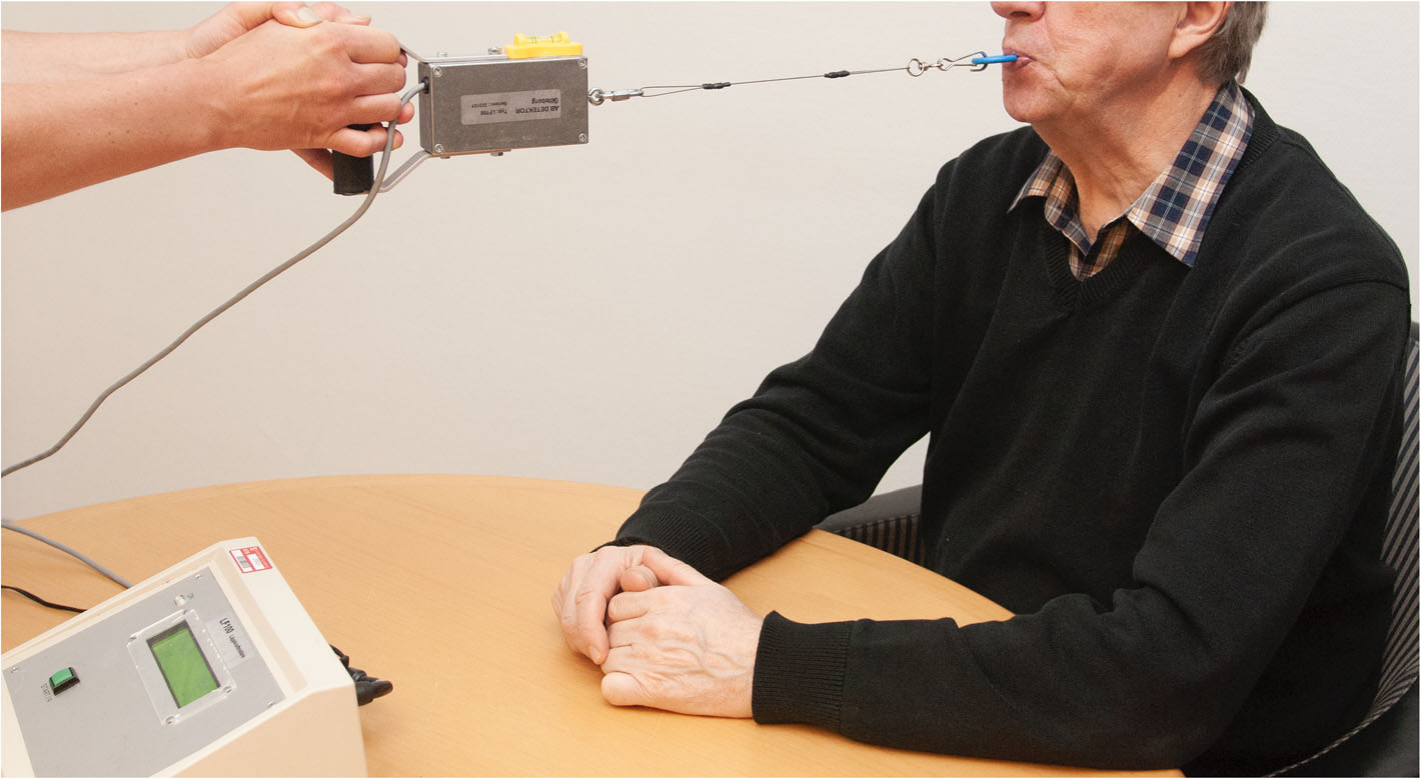
The lip force meter, LF100 (MHC1 AB Detector, Gothenburg, Sweden) (own image). LF100 is a device for measuring the force (in newtons) of the buccinators. The handle on the LF100 is connected to the oral device

Oropharyngeal dysphagia (symptoms of swallowing dysfunction related to the oral cavity and/or pharynx) is one of the most common complications in acute stroke [1]. Traditionally, dysphagia has been managed by compensatory strategies: for example, postural adjustments, swallowing manoeuvres, or modifying the consistency of food/liquid. However, during the late 1990s, there was increasing interest in caring for and improving swallowing function in stroke patients with dysphagia. Orofacial regulation therapy was introduced in 1990 [2], especially as therapy for children with oropharyngeal dysphagia. It comprises orofacial sensory-vibration stimulation with an electric toothbrush [3] or manual massage, sometimes in combination with active sensorimotor training with various oral appliances such as a palatal plate [4] or an oral neuromuscular training device [5]. In 2008, the Muppy® oral device (see Fig. 1) was used to measure lip force with a newton meter (see Fig. 2), and the study showed a significant difference in lip force between healthy subjects and stroke-affected patients with swallowing dysfunction [6]. Lip force and swallowing function are shown to depend on the same complex neuromuscular activity [7]. The same activity that initiates a swallow is activated when lip-force is measured [8]. This suggested that patients with dysphagia could use an oral device to self-train their weak oral and pharyngeal muscles; that is, for neuromuscular training. Improvement in swallowing function has been reported among older people with dysphagia after oral neuromuscular training [9]. Until now, no randomized, controlled study has been performed to investigate the effect of oral neuromuscular training as treatment for post-stroke dysphagia. This study aimed to determine whether adding oral neuromuscular training to orofacial sensory-vibration stimulation improves swallowing function in participants with swallowing dysfunction after a stroke. The secondary aim was to assess how well the results of the timed water-swallow test (TWST) [10–12] correspond with findings of swallowing dysfunction from videofluoroscopy (VFS).

## Methods

### Study design and setting

This was a two-centre study with an intention-to-treat, prospective, randomized, open-label trial with blinded evaluators (PROBE) design. Information on the study design can be found at ClinicalTrials.gov, identifier: NCT04164420. The study was conducted according to the recommendations for Interventional Trials and the CONSORT guidelines for randomised controlled trials (Additional file 1). Patients with a first-ever stroke and persistent swallowing dysfunction 4 weeks post-stroke according to the timed water-swallow test (TWST) [10–12], treated at Umeå University Hospital or Hudiksvall Hospital, Sweden, were offered to participate in the interventional study. The participants were randomized into an intervention group that received oral neuromuscular training for 5 weeks or a control group. A randomization list was constructed by the statistician and trial manager and was stored in envelopes locked in a cupboard. The randomization list did not include any stratification or minimization. For ethical reasons, both groups continued to receive orofacial sensory-vibration stimulation in parallel, initiated before inclusion, if the patients showed dysphagia 1 week after their stroke. Evaluations were made at baseline (4 weeks after stroke onset), at the end of treatment (after 5 weeks treatment), and at the 12-month follow-up after a period without treatment to determine if any lasting positive training effect was present. At the end of treatment and 12-month follow-up, the evaluating assessors were blinded to what treatment the participants had received.

### Participants

Inclusion criteria for participation were the following: first-time stroke and a pathological TWST [10–12] 4 weeks after stroke onset. Exclusion criteria were: inability to cooperate, percutaneous endoscopic gastrostomy (PEG), neurological diseases other than stroke, known history of dysphagia prior to the stroke, prominent horizontal overbite (contra-indication due to the oral device’s design), or hypersensitivity to the acrylate.

### Treatment

#### Oral neuromuscular training

An oral device (Muppy®; Dr. Hinz Dental, Herne, Germany) was used for oral neuromuscular training (see Fig. 1) that aims to stimulate sensory input and strengthen the facial, oral, and pharyngeal muscles [13]. During the training, the device was placed pre-dentally behind closed lips, and the patient sat with the body and head in a strictly upright position. The participants were instructed to hold the device against a gradually-increasing horizontal pulling force for 5–10 s, whilst trying to resist the force by tightening the lips [13]. The participants were instructed to achieve a pulling force that was as high as possible without losing hold of the device with the lips. The exercise was performed three times per session, and three times daily before eating. If the participant was unable to hold the oral device, relatives or ward staff were instructed to assist in providing traction at a right angle to the mouth. Training was also possible when sitting in a bed with the head supported firmly. Verbal, practical, and written instructions about training at home were given to the participants, relatives, or care assistants. The participant or care giver reported the training in a log book that was evaluated after a 5-week training period. The log book was placed visible for the participant and care givers as a reminder of keeping up the training, and it was considered reliable.

#### Orofacial sensory-vibration stimulation

**Fig. 3:**
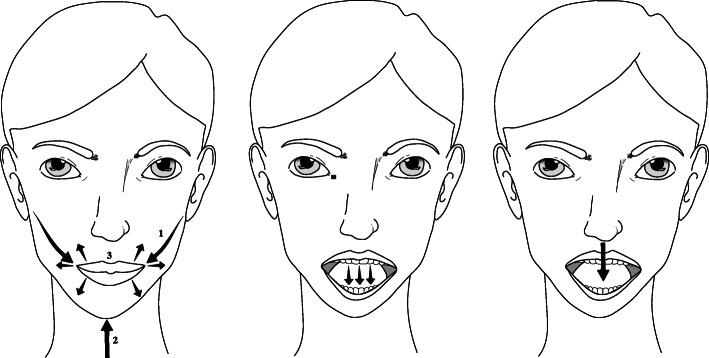
Orofacial sensory-vibration stimulation using an electric toothbrush. (Left) Stimulation of (1) the buccinator mechanism that activates tongue retraction; (2) the muscles of the floor (m. digastricus anterior abdomen, m. mylohyoideus and m. geniohyoideus) that will lift the hyoid bone forward and upward and activate the swallow reflex; and (3) the lips, enhancing lip closure. (Middle) Stimulation of the front of the tongue that activates and raises the root of the tongue, thus passively activating the receptors in the anterior faucial arch to perform the swallowing reflex and indirectly lift the velum. (Right) Stimulation of the tip of the tongue by downward pressure improves the force of the tongue. All the different movements in the left, middle, and right images also provide sensory stimulation (through n trigeminus, afferent pathway) and feedback as a motor response (through n facialis, efferent pathway), that describe the sensory-motor reflex arc. Illustrations: Anna Jäghagen (own illustration/image)

All participants in the intervention group and the control group self-administered or were assisted by relatives or ward staff in orofacial sensory-vibration stimulation (see Fig. 3) by using an electric toothbrush with a round and rotating head (Oral-B®, Procter & Gamble, Cincinnati, USA) [3]. Instructions were given on how to stimulate the buccinator mechanism [4, 7], lips, external floor, and the tongue, three times daily before a meal.

### Outcome

The primary outcome was changes in swallowing rate measured by TWST [10–12] at the end of treatment compared with baseline and 12 months post-treatment, within each group and comparing the intervention group with the control group. Secondary outcomes were changes in lip force measured by a lip-force test [6] at the end of treatment compared with baseline and 12 months post-treatment, comparing the intervention group with the control group. Swallowing dysfunction according to VFS, including the presence of aspiration-penetration as sign of unsafe swallowing as well as premature spillage and pharyngeal residue as signs of ineffective swallowing, was assessed for comparison with the TWST results.

### Procedure

**Fig. 4:**
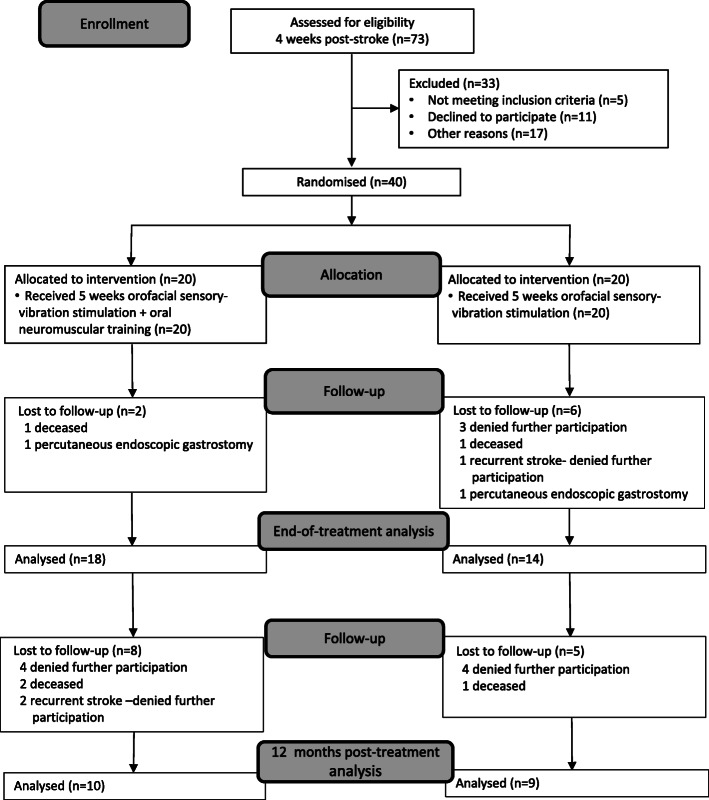
Flowchart of the subject inclusion and data collection processes

For both study groups, the swallowing rate (TWST), lip force, and swallowing function (VFS) were assessed. A nurse, speech-and-language pathologist, or physician performed the assessments, except the VFS examinations, which were performed by a general or an oral-and-maxillofacial radiologist. A flow-chart of the included participants and the study process is shown in Fig. 4.

#### Timed Water-Swallow Test (TWST)

The participant first received a teaspoon of water, repeated three times consecutively. In case of a total inability to swallow, or if a swallow was misdirected (e.g., there were clinical signs of aspiration such as cough or wet/gurgly voice), the swallowing rate was graded as zero and the TWST was not performed. If the swallowing was safe during the teaspoon test, a TWST was completed.

The TWST is a timed test of a person’s ability to swallow water [10–12]. It was performed with the participants sitting in upright position. The participants were instructed to swallow water (150 mL) as quickly as possible without pausing. The time was recorded from the start of drinking until the last swallow was completed. Any remaining water in the glass was measured. The swallowing rate was calculated as the amount of swallowed water (in millilitres) divided by time (seconds). A swallowing rate of 10 mL/s was regarded as the lower normal limit [10–12]. The TWST has been demonstrated to have high intra-rater, inter-rater, and test-retest reliability and to be a valid index for assessing swallowing dysfunction [10–12, 14].

#### Lip-force test

Lip force was recorded in newtons using a LF100 (MHC1 AB Detector, Gothenburg, Sweden) [6] (see Fig. 2). The patient was instructed to hold a pre-dentally placed oral device (Muppy®) (see Fig. 1) for as long as possible by tightening the lips and pressing the head backwards as the investigator applied traction at a right angle to the patient’s mouth with increasing force for 10 s, or until the subject lost their grip of the oral device. Each subject was measured three times, with a 2-min rest between attempts. The maximum lip-force value was recorded and blinded for the investigator. The lower limit for normal lip force was 15 N [6].

#### Videofluoroscopy (VFS)

During VFS in lateral projection, the participant initially swallowed 5 mL of iodine contrast medium (Visipaque® 320 mg I/mL) delivered with a spoon, to assess the presence of aspiration (before, during, or after swallowing). If severe aspiration was present, the examination was concluded. If no or minor aspiration was found, a bolus of 10 mL iodine contrast medium was tested. If there was no aspiration, the examination proceeded with 10 and 20 mL liquid barium sulphate contrast medium (Mixobar® Colon 1 mg/mL) delivered with a spoon, and a free amount of liquid barium sulphate from a cup. If the patient was still free of aspiration, this was followed by assessment in the frontal projection with 5 mL, 20 mL, and a free amount of barium sulphate contrast medium. To assess correspondence with TWST, oral and pharyngeal residue (bolus retention after the bolus has passed through the pharynx), premature spillage (bolus entering the pharynx without eliciting the swallowing reflex, with the airway remaining open with increased risk of aspiration), penetration (bolus entering the larynx and proceeding below the vocal cords), and aspiration (bolus entering the airway below the vocal cords) with and without cough were recorded. The improvement in swallowing function was assessed according to the penetration-aspiration scale (the PAS is scored 1–8, where score 1 means no bolus enters the airway, and scores 2–8 are different degrees of penetration or aspiration into the airway) [15]. The PAS scores were dichotomised into ≤2 versus > 2. All assessments were performed individually by two radiologists. The inter-rater agreement was calculated. The assessments were compared, and, in case of disagreement, consensus was reached in a joint reassessment that was used as the final result.

### Statistical analyses

A professional statistician and a data manager from the clinical research centre at Uppsala University were involved in planning the study according to good clinical practice (GCP). In order to detect a critical change with a power of 80%, a sample size of 44 participants (22 in the intervention group and 22 in the control group) that fulfilled the study protocol was required for a type I error of 5%. Wilcoxon’s signed-rank test was used to evaluate possible changes within a group between different test occasions. Spearman’s rank test was used for correlation analysis of different test methods. The Mann-Whitney *U* test and Fisher’s exact test were used to compare data from the intervention group and the control group. When non-parametric tests were used, confidence intervals are presented to indicate the precision of the estimates. Inter-rater reliability was estimated using Cohen’s Kappa (κ). A value of *P* < 0.05 was considered significant. All statistical analyses were performed using SAS version 9.1 (SAS Institute Inc., Carey, NC, USA).

## Results

### Participants

At 4 weeks post-stroke, 73 persons who had been identified with dysphagia one-week post-stroke were screened for persisting oropharyngeal swallowing dysfunction measured with TWST. Of these, 17 persons had regained normal swallowing function and were excluded, and 51 persons showed dysfunction, 11 of whom declined to participate. The remaining 40 participants were randomly assigned to either the intervention group (*n* = 20, of who 9 were women; mean age 75 years, range 60–85) or the control group (*n* = 20, of who 6 were women; mean age 75 years, range 56–90) (See Fig. 4). Characterization of the participants and stroke lesion is shown in Table 1. For a further detailed description of the participants’ characterization, see Additional file 2. After the treatment period, 32 participants (80%) were alive and available for analysis, and at the 12-month follow-up 19 participants (47.5%) were alive and available for analysis (see Fig. 3). The study was stopped somewhat prematurely due to a slow inclusion rate.

**Table 1:** Baseline demographic and clinical characteristics

Variable	Control group *n* = 20	Intervention group *n* = 20
Age	75 [56–90]	75 [60–85]
Sex		
Male	14 (70)	11 (55)
Female	6 (30)	9 (45)
Stroke type		
Ischemic	16 (80)	16 (80)
ICH	3 (15)	4 (20)
Ischemic and ICH	1 (5)	–
Left hemisphere	6 (30)	7 (35)
Right hemisphere	10 (50)	10 (50)
Supratentorial	15 (75)	16 (80)
Infratentorial	3 (15)	4 (20)
Supra- and infratentorial	1 (5)	–
Lowered consciousness at hospital admission	6 (30)	6 (30)

Data are presented as n (%) or mean [range]

*Abbreviation*: *ICH* Intracerebral hemorrhage

### Swallowing rate

**Fig. 5:**
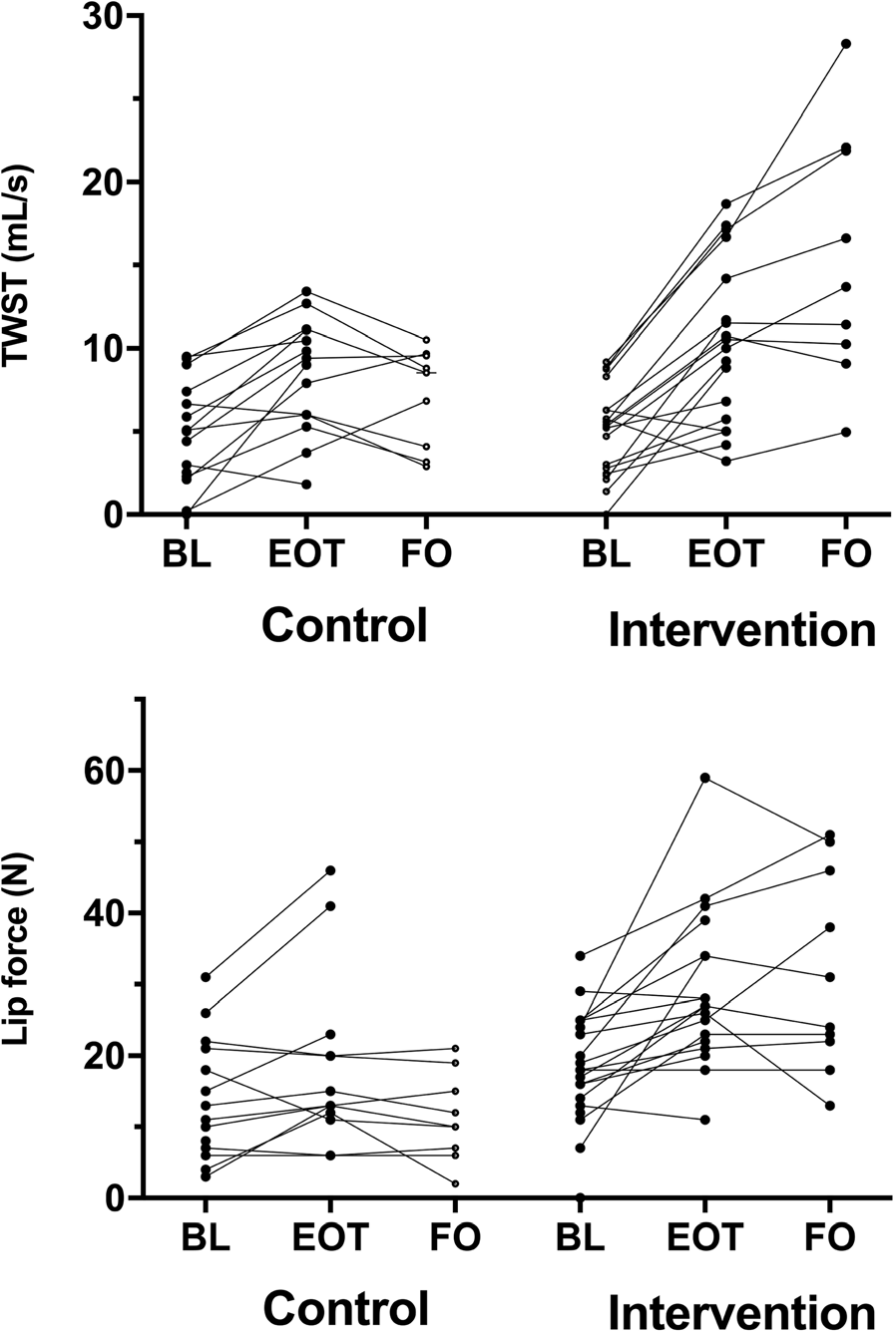
Line plots indicating the individual changes in swallowing rate measured by the timed water-swallow test (TWST) and in lip force measured in newtons (N), from baseline (BL) to the end of treatment (EOT) and follow-up 12 months after completed treatment (FO) for the control group and the intervention group

At the end of treatment, the intervention group and control group showed similar improvement in swallowing rate compared with baseline (*P* < 0.001 and *P* = 0.001, respectively; Table 2) without any significant difference in improvement between the two groups (*P* = 0.133; Table 2). At 12 months post-treatment, the swallowing rate had improved significantly more in the intervention group compared with the control group (*P* = 0.032; Table 2). Individual line plots for changes in swallowing rate between time points are depicted in Fig. 5.

**Table 2:** Analysis of swallowing rate, lip force and VFS immediately after treatment and 12 months post-treatment

Outcome	Control	Within-group differences		Intervention	Within-group differences		Between-groups differences	No. subjects
	Median (ranges)	(95% CI)	*P* value	Median (ranges)	(95% CI)	*P* value	*P* value	Control/Intervention
Swallowing rate^a^								
Baseline	5.1 (0.0–9.5)			5.3 (0.0–9.2)				20/20
End of treatment (5 weeks)	9.2 (1.8–13.4)	3.6 (1.0–5.8)	0.001^b^	10.3 (3.2–18.7)	5.3 (2.2–8.6)	< 0.001^b^	0.133^c^	14/18
12 months post-treatment	8.5 (2.9–10.5)	1.1 (−1.0–6.6)	0.164^b^	13.7 (5.0–28.3)	9.0 (3.7–13.6)	0.008^b^	0.032^c^	9/9
Lip force								
Baseline	14.0 (3.0–31.0)			18.5 (7.0–34.0)				14/18
End-of-treatment (5 weeks)	14.0 (6.0–46.0)	2 (−1–10)	0.079	27.0 (11.0–59.0)	7 (3–13)	< 0.001	0.066	14/18
12 months post-treatment	10.0 (2.0–21.0)	−1 (−3–1)	0.328	31.0 (18.0–51.0)	12 (4–26)	0.008	0.001	9/9
VFS (PAS score)								
Baseline	6 (1–8)			3 (1–8)				13/18
End of treatment (5 weeks)	1 (1–8)	0.0 (−6–6)	0.524^b^	2 (1–8)	0.0 (−2–0)	0.219^b^	0.999	11/18
12 months post-treatment	1.5 (1–8)	0.0 (−7–6)	0.276^b^	2 (1–8)	−0.5 (−3–0)	0.058^b^	0.912	8/9

*Abbreviations*: *VFS* videofluoroscopic evaluation of swallowing, *PAS* Penetration Aspiration Scale, *CI* confidence interval, *TWST* timed water-swallow test

^a^According to TWST. Normal swallowing rate is ≥10 mL/s, whereas a rate < 10 mL/s indicates swallowing dysfunction. An increased rate indicates improvement of swallowing function

^b^Wilcoxon signed-rank test

^c^Mann Whitney *U*-test

### Lip force

At the end of treatment, the intervention group showed an improvement in lip force (*P* < 0.001; Table 2), whereas the control group showed non-significant improvement in lip force (*P* = 0.079; Table 2). There was no significant between-group difference at the end of treatment (*P* = 0.066; Table 2). At 12 months post-treatment, lip force was significantly improved in the intervention group compared with the control group (*P* = 0.001; Table 2). Individual line plots for changes in lip force between time points are depicted in Fig. 5.

### Videofluoroscopy

At baseline, 32 of the 40 participants had available VFS. The remaining 8 were missing for logistical reasons. All 32 participants showed swallowing dysfunction at baseline, with PAS > 2 in 53% (intervention group, 50%; controls, 57%), premature spillage in 78% (intervention group, 72%; controls, 86%), and pharyngeal retention in 78% (intervention group, 83%; controls, 71%), see Table 3. Thus, there was good correspondence between the TWST score < 10 mL/sec and the VFS findings. There was no significant difference in improvement between the two groups at any of the three test occasions in any of the VFS parameters. The inter-rater reliability varied from near perfect agreement to perfect agreement for all measurements: premature spillage (κ = 0.93), pharyngeal retention (κ = 0.81), aspiration (κ = 1.00), and PAS (κ = 0.93).

**Table 3:** Swallowing dysfunction at baseline according to the VFS

Baseline	Control *n* = 14	Intervention *n* = 18	*P*-value
Premature spillage			0.426^a^
Yes	12 (86)	13 (72)	
No	2 (14)	5 (28)	
Retention pharynx^*^			0.669^a^
Yes	10 (71)	15 (83)	
No	4 (29)	3 (17)	
Aspiration			0.178^b^
Yes	8 (57)	6 (33)	
No	6 (43)	12 (67)	
PAS > 2			0.688^a^
Yes	8 (57)	9 (50)	
No	6 (43)	9 (50)	
One or more dysfunction			NA
Yes	14 (100)	18 (100)	
No	0 (0)	0 (0)	

Data are given as n (%)

*Abbreviations*: *VFS* videofluoroscopy, *PAS* Penetration Aspiration Scale, *NA* Not Applicable

^*^Retention pharynx refers to retention in the vallecula or piriform sinus

^a^Fisher’s exact test

^b^Chi-squared test

### Adverse events

No adverse events were registered in the intervention or the control group.

## Discussion

This randomized controlled trial with blinded evaluators showed that oral neuromuscular training improved swallowing function in the long term among people with post-stroke dysphagia. While there were no significant between-group differences at the end of treatment in swallowing rate, lip force, or aspiration-penetration on VFS, the intervention group showed a significant improvement in swallowing rate and lip force at 12-month follow-up compared with the control group. These findings indicate the good effects of oral neuromuscular training as a treatment option for post-stroke dysphagia. There is, however, a need for larger randomized controlled trials to confirm our preliminary, positive long-term results.

Both the intervention and control groups showed significant within-group improvements in swallowing rate after the 5-week training period, but there was no significant between-group difference. The non-significant between-group difference may be due to several factors. One is that both groups received active rehabilitation (toothbrush stimulation) during the treatment period and with positive outcome, indicating that both treatments had an immediate effect on swallowing. However, only the neuromuscular training was found to have a long-term effect. Another factor was the small sample size, indicating that there could be a lack of sufficient statistical power to generalise the conclusions of the findings. Yet another factor could be the spontaneous remission of swallowing dysfunction that is usually observed within 2–3 weeks after the stroke incident [16, 17]. In this study the participants were recruited 4 weeks after the stroke event, making it difficult to determine whether all the improvement in the swallowing rate was due to the therapeutic intervention or, to some extent, to spontaneous recovery.

However, the lasting improvement in swallowing rate at 12 months post-treatment in the intervention group compared with the control group indicates that oral neuromuscular training may be more effective in triggering the reorganisation of the brain [18] than orofacial sensory-vibration stimulation alone. In contrast to orofacial sensory-vibration stimulation, oral neuromuscular training with an oral device aims to both stimulate sensory input and strengthen the facial, oral, and pharyngeal muscles [13].

Exercised-based approaches to swallowing rehabilitation have been shown to not only improve swallowing function in people with dysphagia, but to improve oral diet and reduce aspiration and dependence on tube feeding [19–22]. Furthermore, exercises to strengthen the swallowing musculature improve swallowing and reduce dysphagia-related comorbidities [19, 21]. In the present study, swallowing function was assessed and improved after oral neuromuscular training, indicating that combined sensorimotor exercise might be a more beneficial swallowing rehabilitation method in the long term than sensory stimulation alone. However, we did not evaluate the effect of oral neuromuscular training on dysphagia-related comorbidities such as malnutrition, dehydration, or pneumonia. Further studies should include these aspects and include swallowing-related quality of life as outcomes when evaluating oral neuromuscular training effects on swallowing dysfunction. Thus, in order to evaluate the full benefit of oral neuromuscular training as a swallowing rehabilitation method.

Improvement in lip force was observed in the intervention group both after 5 weeks of oral neuromuscular training and at the 12-month follow-up, whereas no significant improvement was observed in the control group at any time. A significant between-group difference was observed only at the 12-month follow-up. These findings, combined with the improvements in swallowing rate, seem to support the finding of previous studies [8, 13] in which oral neuromuscular training improved both swallowing rate and lip force.

When used to identify swallowing dysfunction, results from TWST proved to have excellent correlation with findings from VFS, in accordance with a previous study [14]. All participants showed dysfunction, either with penetration/aspiration of contrast medium entering the airway and causing unsafe swallowing at baseline, or with premature spillage and/or pharyngeal residue revealing ineffective pharyngeal swallowing. It is essential to have valid and reliable screening tools in clinical settings since gold standard assessments (VFS and videoendoscopy) are not always available or feasible to implement due to both social and economic conditions, as well as the existing health policies. The advantage of TWST is that the tool can easily be used as a quantifiable measure of treatment effect (mL/s before and after treatment). The disadvantage is that the tool only assesses the swallowing function using liquid bolus. Other potential screening tools for swallowing assessment that includes tests of semi-solid and solid texture is, e.g., the Gugging Swallowing Screen (GUSS) [23] or the volume-viscosity swallow test (V-VST) [24].

The present study is the first randomized controlled trial of oral neuromuscular training with an oral device as post-stroke dysphagia treatment and in which blinded assessors were used at the end of treatment and at 12-month follow-up. However, it does have some methodological issues. A common challenge with clinical trials, including this study, is to recruit enough participants to obtain acceptable statistical power at follow-up, after drop-out. The number of participants included at baseline almost reached the number targeted by the power analyses, but many participants were lost at follow-up due to mortality, PEG, recurrent stroke, or because they declined continued participation (see Fig. 3 and Additional file 2). Another limitation is that the randomization to either the intervention or control group was based on TWST results and not on VFS findings. Since the randomization procedure did not take into account important prognostic factors at baseline, an uneven distribution of aspiration at baseline (33% in the intervention group and 57% among controls) might have contributed to the lack of significant outcome on differences in improvement according to VFS. It might have been easier to improve in PAS in the control group with higher frequency of aspiration at baseline than in the intervention group. Thus, an objective finding on the severity of swallowing dysfunction may be an important prognostic factor in the participants’ ability to improve the impaired function, and should be considered at randomisation.

## Conclusions

Oral neuromuscular training in participants with impaired swallowing rate after stroke had no significant effect on swallowing rate immediately after the 5-week training but resulted in long-term improvement in swallowing function. TWST findings corresponded with VFS findings and TWST therefore appears to be a feasible method with which to identify persons with swallowing dysfunction after stroke. Larger randomized controlled trials are required to confirm the preliminary positive long-term results on swallowing function in this study.

## Supplementary Information

**Additional file 1.**

**Additional file 2.** Participants' characteristics

## Data Availability

The datasets generated and/or analysed during the current study are not publicly available due to institutional restrictions but are available from the corresponding author on reasonable request.

## Abbreviations

TWST: Timed water-swallow test
VFS: Videofluoroscopy
PAS: Penetration-aspiration scale
