# Supplementary material for: Oral neuromuscular training in patients with dysphagia after stroke: a prospective, randomized, open-label study with blinded evaluators
Source: BMC Neurol. 2020 Nov 7;20:405. doi: 10.1186/s12883-020-01980-1 (PMC7648322; doi:10.1186/s12883-020-01980-1)
Supplement: Supplementary file 2 — Additional file 2. Participants' characteristics [file 12883_2020_1980_MOESM2_ESM.pdf]

Appendix 2. Participants' characteristics

Intervention / control	Sex	Age (range)	Admission symptoms (except dysphagia)	Brain CT/MR	Stroke type	Stroke lesion location right (R)/ left (L)	Stroke lesion location (supra- /infra- tentorial)	EoT	12 m	Reason for loss of follow-up
I1	F	40-69	Right hemiparesis, lowerd consciousness (RLS 2)	CT acute infarction left frontoparietal lobe	IS	L	ST	x	-	DFP
I2	M	40-69	Wallenberg syndrome; Horner, dysarthria, sensory loss	MR acute ischemia medulla oblongata	IS	N.A.	IT	x	-	DFP
I3	M	70-89	Left hemparesis, lowerd consciousness (RLS 2), dysarthria, NIHSS 22	CT acute infarction right MCA territory	IS	R	ST	x	-	Death
I4	M	70-89	Headache, confusion	CT acute iCH frontal lobe	ICH	R	ST lobar	x	x	
I5	M	70-89	Left hemiparesis, dysarthria, NIHSS 7	CT unremarkable	IS	R	ST	x	x	
I6	M	70-89	Left hemiparesis, dysarthria	CT unremarkable	IS	R	ST	x	x	
I7	F	70-89	Right hemiparesis, aphasia	CT left MCA infarction frontal lobe	IS	L	ST	x	x	
I8	M	70-89	Left hemiparesis, dysarthria, NIHSS 7,	CT right deep ICH	ICH	R	ST deep	-	-	Death
I9	M	40-69	Rotational vertigo, nasuea, sensory loss left side, ataxia	CT acute brain stem and cerebellar infarction	IS	L	IT	x	x	
I10	F	70-89	Right hemiparesis, lowered consciousness (RLS 3)	CT left MCA infarction	IS	L	ST	x	-	Recurrent stroke, DFP
I11	M	40-69	Left hemiparesis, hemianopia, dysarthria, sensory loss tongue	CT unremarkable	IS	R	ST	x	x	
I12	M	70-89	Left hemiparesis, conjugated eye deviation, drowsy (RLS 2), dysarthria	CT unremarkable	IS	R	ST	x	-	Death
I13	M	40-69	Left hemiparesis	CT unremarkable	IS	R	ST	x	x	
I14	M	40-69	Right hemiparesis, conjugated eye deviation, lowered consciousness (RLS 2), NIHSS 25	CT left MCA infarction	IS	L	ST	x	x	
I15	M	40-69	Left hemiparesis, dysarthria, NIHSS 5	CT right MCA lacunar infarction	IS	L	ST	x	x	
I16	K	70-89	Vertigo, nausea, dysarthria	MR right cerebellar infarction	IS	L	IT	x	-	DFP
I17	M	40-69	Left hemiparesis, dysarthria, lowerd consciousness (RLS 2-3)	CT right ICH 70 ml with intraventricular extension	ICH	R	ST deep	x	-	PEG
I18	M	70-89	Left hemparesi and sensory loss; dysarthria, NIHSS 11	CT right MCA infarction frontotemporal lobe	IS	R	ST	x	x	
I19	F	70-89	Left hemiparesis and sensory loss, diplopia, dyscoordination, conjugated eye deviation	CT brain stem ICH	ICH	L	IT	x	-	DFP
I20	F	70-89	Aphasia, dysarthria, right facial paresis; NIHSS 3	CT left MCA infarction frontoparietal lobe	IS	L	ST	x	-	Recurrent stroke, DFP
C1	F	70-89	Left hemiparesis and sensory loss, dysarthria, rotational vertigo	CT unremarkable	IS	R	IT	x	x	
C2	M	70-89	Left hemiparesis, lowered consciousness (RLS 2), dysarthria, NIHSS 19	CT right MCA infarction	IS	R	IT	x	x	
C3	F	40-69	Left hemiparesis, dysarthria, NIHSS 4	CT right deep ICH (thalamus)	ICH	R	ST deep	-	-	DFP
C4	M	40-69	Acute vertigo	CT ICH left pons and cerebellum	IS	R	IT	x	x	
C5	M	70-89	Left hemiparesis, dysarthria	CT right deep ICH	IS	R	ST	x	x	
C6	F	40-69	Left hemiparesis, dysarthria, NIHSS 5	CT bilateral lacunar infarcts in the basal ganglia	IS	R	ST	x	-	DFP
C7	M	40-69	Left hemiparesis, lowered consciousness (RLS 2), dysarthria	CT right MCA infarction	IS	R	ST	x	x	
C8	M	70-89	Right hemiparesis	CT cerebellar ICH followed by left MCA infarction	ICH + IS	L	IT + ST	-	-	Recurrent stroke, DFP
C9	M	40-69	Right hemiparesis, aphasia, tongue deviation	CT left MCA infarction	IS	L	ST	x	x	
C10	M	70-89	Right hemiparesis, aphasia	CT large left MCA infarction	IS	L	ST	-	-	Death
C11	M	70-89	Left hemiparesis	CT right MCA infarction	IS	R	ST	x	x	
C12	F	70-89	Left hemiparesis, diplopia, dysarthria	CT unremarkable	IS	R	IT	x	x	
C13	F	70-89	Left hemiparesis, dysarthria, NIHSS 2	CT right MCA infarction	IS	R	ST	x	x	
C14	F	70-89	Left hemiparesis, lowered consciousness (RLS 3), NIHSS 29	CT right MCA infarction	IS	R	ST	x	-	DFP
C15	F	70-89	Left hemiparesis, dysarthria, lowered consiousness (RLS 2-3), NIHSS 20	CT right deep ICH (basal ganglia)	ICH	R	ST deep	x	-	DFP
C16	M	90+	Left hemiparesis, dysarthria, aphasia	CT left MCA infarction	IS	L	ST	-	-	PEG
C17	F	70-89	Right hemiparesis, neglect, lowered consciousness (RLS 2-3) NIHSS 17	CT unremarkable	IS	L	ST	x	-	DFP
C18	F	70-89	Right hemiparesis, NIHSS 5	MR right MCA infarction	IS	R	ST	x	-	DFP
C19	M	70-89	Right hemiparesis, dysarthria	CT left deep ICH (thalamus)	ICH	L	ST deep	x	x	
C20	M	70-89	Left hemiparesis, dysartria	CT unremarkable	IS	R	ST	x	-	DFP

Abbrevations: NHISS = National Institutes of Health Stroke Scale; CT = Computed tomography; MR = Magnetic resonance; MCA = Middle cerebral artery; EoT = End of treatment; 12 m = 12 months follow-up; IS = Ischemic stoke; ICH = Intracerebrlal hemorrhage; ST = Supratentorial; IT = Infratentorial; DFP = Denied further participation; PEG = Percutaneous endoscopic gastrostomy

	Date of birth	Initials	Date of onset	Sex	Age
M2	19270919-	SN	2006-11-28	F	69
M4	19470406-	SB	2007-03-03	M	60
M5	19320629-	AS	2007-10-23	M	75
M8	19251207-	IS	2008-04-29	M	82
M10	19261204-	TN	2009-05-13	M	82
M11	19231213-	RW	2009-05-30	M	85
M14	19320628-	EL	2010-01-11	F	78
M16	19270828-	EO	2010-01-09	M	83
M18	19441231-	ÅE	2010-03-16	M	65
M20	19300116-	BB	2010-06-09	F	80
M22	19411001-	GS	2010-10-11	M	69
M24	1920130-	SJ	2011-01-29	M	79
M25	19470221-	JEB	2011-01-05	M	63
M27	19460527-	SB	2011-09-27	M	65
M29	19420111-	BS	2011-11-05	M	69
M32	19330922-	KÅ	2012-09-18	K	79
U01	19300707-	TP	2011-08-11	M	82
U04	19320419-	MB	2011-10-25	F	79
U06	19380609-	IB	2013-03-12	F	74

C1	19350826-	IJ	2006-10-28	F	71
C3	19360520-	BB	2007-07-12	M	70
C7	19510630-	OB	2008-01-19	M	56
C9	19240704-	SS	2009-03-31	M	85
C13	19510518-	NG	2010-02-10	M	58
C17	19500105-	KA	2010-02-16	M	60
C21	19300811-	KN	2010-10-08	M	80
C23	19260208-	GW	2010-10-25	F	84
C26	19311213-	GL	2011-09-08	F	79
C30	19361005-	GW	2011-12-29	F	75
C33	19351209-	VM	2012-09-23	F	76
C35	19330823-	GL	2012-12-26	F	79
U02	19281110-	RS	2011-08-30	M	82
U07	19401221-	JI	2013-09-03	M	72

Previous/concommitant diseases
TIA
Hyperlipidemia
Hypothyreosis
AF, warfarin treated
Stroke left sequele, paroxysamal AF
Op aortic aneurysm, prostata-ca
Osteoporosis, hyperpara, COPD
COPD, st p stroke 15-y ago no sequele, HT,
HT, AF
Glaucoma, AF
DM-2
Prostata-neo, HT, AF
Stroke x 3, HT, hyperlipidemia, DM-2, AF
MI, AP
DM-2, HT, TIA
TIA x 3, hyperlipidemia
MIx2; CABG; op aortic aneurysm; CKD stage
AF, warfarin, HT, status post ICH
HT, dyslipedemia, st post IS, DM

Urinary bladder neoplasm
HT, AP, AF
HT, Mb Crohn
DM-2
HT, gout, social fobia
HT, DM-2, AF
Prostata-neo, DM-2
AF, COPD, trigeminus-neuralgia
HT
HT, st post AMI, AP
Hypothyr, status post mammar-ca, depre
Post-polio syndr with vä HP, AF
HT, dyslipidemia, DM-2,
HT, hyperlipidemia, DM-2, st p IS 2001

Admission symptoms (except dysphagia)
Right hemiparesis, drowsy (RLS 2), aphasia
Wallenberg syndrome with Horner, dysarthria, sensory loss right abdomen and right ar
Left hemparesis, slightlys drowsy (RLS 2), NIHSS 22; aphasia, dysarthria
Headache, confusion
Left hemiparesis, dysarthria, NIHSS 7
Left hemiparesis, dysarthria
Right hemiparesis, aphasia
Left hemiparesis, NIHSS 7, dysarthria
Rotational vertigo, nasuea, sensory loss left side, ataxia
Right hemiparesis, lowered consciousness (RLS 3)
AMI (NSTEMI), during PCI left hemiparesis, hemianopia, dysarthria, sensory loss tongue
Left hemiparesis, conjugated eye deviation, drowsy (RLS 2), dysarthria
Left hemiparesis
Right hemiparesis, conjugated eye deviation, NIHSS 25
Left hemiparesis, dysarthria, NIHSS 5
Vertigo, nausea, dysarthria
Left hemparesi and sensory loss; dysarthria, neglect, NIHSS 11
Left hemiparesis and sensory loss, diplopia, dyscoordination, conjugated eye deviation
Aphasia, dysarthria, right facial paresis; NIHSS 3

Left hemiparesis and sensory loss, dysarthria, rotational vertigo
Left hemiparesis, slightly drowsy (RLS 2), dysarthria, NIHSS 19
Acute vertigo
Left hemiparesis, dysarthria
Left hemiparesis, slightly lowered consciousness (RLS 2), dysarthria
Right hemiparesis, aphasia, tongue deviation
Left hemiparesis
Left hemiparesis, diplopia, dysarthia
Left hemiparesis, dysarthria, NIHSS 2
Left hemiparesis, dysarthria, NIHSS 20
Right hemiparesis, neglec, NIHSS 17
Right hemiparesis, NIHSS 5
Right hemiparesis, dysarthria
Left hemiparesis, dysartria,

Stroke lesion (Infarction/ICH; left/right hemisphere; supra-/infratentorial)		Acute treatment
IS, left, supratentorial	Left MCA Infarction	
IS, infratentorial	Brain stem infarction	
IS, right, supratentorial	Right MCA infarction	Thrombolysis
ICH, right lobar	Right lobar ICH	Reversal antiocoagula
IS, right, supratentorial	Right MCA infarction	
IS, right, supratentorial	Right MCA infarction	
IS, left, supratentorial	Left MCA Infarction	Thrombolysis
ICH, right deep	Right deep ICH	
IS, left, infratentorial	Left brain stem and cerebellar infarction	
IS, left, supratentorial	Left MCA Infarction	
IS, right, supratentorial	Right MCA infarction	
IS, right, supratentorial	Right MCA infarction	Thrombolysis
IS, right, supratentorial	Right MCA infarction	
IS, left, supratentorial	Left MCA Infarction	
IS, right, supratentorial	Right MCA infarction	
IS, left, infratentorial	Right cerebellar infarction	
IS, right, supratentorial	Right MCA infarction	Thrombolysis
ICH, infratentorial	Brain stem ICH	Reversal antiocoagula
IS, left, supratentorial	Left MCA infarction	Thrombolysis
IS, right, infratentorial	Infratentorial ischemia	
IS, right, supratentorial	Right MCA infarction	Thrombolysis
ICH, right, infratentorial	Right brain stem and cerebellar ICH	
ICH, right, deep	Right MCA ICH	
IS, right, supratentorial	Right MCA infarction	
IS, left, supratentorial	Left MCA infarction	
IS, right, supratentorial	Right MCA infarction	Thrombolysis
IS, right, infratentorial	Right infratentorial ischemia	
IS, right, supratentorial	Right MCA infarction	
ICH, right, deep	Right deep ICH	
IS, left, supratentorial	Left MCA infarction	Thrombolysis
IS, right, supratentorial	Right MCA infarction	
ICH, left, deep	Left deep ICH	
IS, right, supratentorial	Right supratentorial ischemia	

Brain CT/MR	Brain CT/MR
CT acute infarction left frontoparietal lobe	CT: Acute infarction left frontoparietal lobe
CT unremarkable; MR acute ischemia medulla o	CT: unremarkable; MR acute ischemia medul
CT acute infarction right MCA territory	CT: färsk inf hö MCA
CT acute iCH frontal lobe	CT: ICH 50ml frontalt hö
CT unremarkable	CT: uva
CT unremarkable	CT: uva
CT left MCA infarction	Gammal ischemi temporo-occ vä, akt först ua
CT right deep ICH	CT: ICH hö
CT acute brain stem and cerebellar infarction	CT uva; 2a us lill-hj-infarkt vä + vä hjärnstam
CT left MCA infarction	CT 2 färka infarkter caps ext vä + parietalt vä
CT unremarkable	CT uva
CT unremarkable	CT uva
CT unremarkable	CT uva
CT left MCA infarction	CT: färsk vä media-infarkt
CT right MCA infarction	CT x 2, 2:a us hö färsk lakunär infarkt
MR right cerebellar infarction	CT uva; MR hö cerebellär infarkt
CT right MCA infarction	CT right frontoparietal infarction 30-50% of M
CT brain stem ICH	CT ICH 6x9x5mm left medulla oblongata (sma
CT left MCA infarction	CT left 4 cm frontoparietal IS
CT unremarkable	CT uva
CT acute right MCA infarction	CT uva; 2:a us hö media-inf
CT ICH left pons and cerebellum	CT ICH vä pons + cerebellum
CT right deep ICH	CT ICH hö MCA
CT right MCA infarction	CT hö parietal inf
CT left MCA infarction	CT x 2; vä färsk inf fronto-temporo-parietalt m
CT right MCA infarction	CT x 2; hö MCA-inf
CT unremarkable	CT x 2 uva, inop elektroder
CT right MCA infarction	CT hö MCA-inf temporo-parietalt
CT right deep ICH	CT ICH hö basala ggl
CT unremarkable	CT x 2; uva
MR right MCA infarction	CT x 2, uva; MR hö inf
CT left deep ICH	CT ICH 7ml left thalamus
CT unremarkable	CT uva; CT-ai left vertebral stenosis

Övr	Med vid utskrivning
	Trombyl, Salures, Seloken, Alvedon
UL halskärl 30	Trombyl, Metformin, Salures, Enalapril, Lipitor, Insulatard
	Trombyl, Levaxin
	Digoxin, Seloken, Enalapril, Simvastatin, Citalopram
	Trombyl, Tambocor, Seloken, Omeprazol
	Trombyl, Felodipin
; 2:a us vä fror	Trombyl, Seloken, Plendil, Simvastatin, Fosamax
	Lasix Ret, Seloken, Enalapril, Simvastatin, Citalopram, KOL-med,
	Trombyl, Metoprolol, Enalapril
	Waran, Seloken,
	Metformin, Glibenklamid, Plavix, Trombyl, Seloken, Enalapril, Simvastatin,
	Waran, Calogen?, Digoxin, Metoprolol, Simvastatin
	Metformin, Daonil, Bisoprolol, Felodipin, Cocaar comp, Simvastatin, Insulat
	Trombyl, Simvastatin, Seloken
	Metformin, Trombyl, Enalapril, Simvasatin, Insulatard, Novmix
	Plavix, Seloken, Simvastatin, Citalopran
ICA; right ICA (	Trombyl, Imdur, Furix, Metoprolol, Felodipin
ll brain stem h	Trombyl, metformin, Impugan, Amoldipin, Enalapril, Simvastatin,
Recurrent IS a	ASA + Clo + Seloken + Atacand + Simvastatin
	Trombyl, Citalopram
	Furix, Suscard, Trombyl, Imdur, Zanidip, Atacand plus, Simvastatin
	Loperamid, Atenolol, Cozaar comp, Citalopram
	Metformin
	Trombyl, Tenormin, Plendil, Atacand Plus, Simvastatin, Ergenyl
ed förskjutnin	Trombyl, Impugan, Metoprolo, Plendil, Enalapril, Simvastatin, Insulatard, H
	Trombyl, Seloken, Enalapril, Simvastatin, Citalopram
	Simvastatin, Trombyl, Digoxin, KOL-med, Citalopram
	Trombyl, Seloken, Atacand
	Salures, Seloken, Atacand, Simvastin,
	Trombyl, Simvastatin, Levaxin, cipralex, Mirtzapin
	Waran, Enalapril,
	Salures, Trombyl, Metformin, Carvedilol, Amlodipin, Atacand, Simvastatin
	Clo, Metformin, Norvasc, Enalapril, Atorvastatin

Insulatard

:ard, Waran

umalog
